# Bicarbonate secretion and acid/base sensing by the intestine

**DOI:** 10.1007/s00424-024-02914-3

**Published:** 2024-02-19

**Authors:** Holger M. Becker, Ursula E. Seidler

**Affiliations:** Department of Gastroenterology, Hannover Medical School, 30625 Hannover, Germany

**Keywords:** CFTR, SLC26a3, SLC26a6, Sodium bicarbonate cotransporter, Intestine, Colon, Cystic fibrosis, Congenital chloride diarrhea, Inflammatory bowel disease

## Abstract

The transport of bicarbonate across the enterocyte cell membrane regulates the intracellular as well as the luminal pH and is an essential part of directional fluid movement in the gut. Since the first description of “active” transport of HCO_3_^−^ ions against a concentration gradient in the 1970s, the fundamental role of HCO_3_^−^ transport for multiple intestinal functions has been recognized. The ion transport proteins have been identified and molecularly characterized, and knockout mouse models have given insight into their individual role in a variety of functions. This review describes the progress made in the last decade regarding novel techniques and new findings in the molecular regulation of intestinal HCO_3_^−^ transport in the different segments of the gut. We discuss human diseases with defects in intestinal HCO_3_^−^ secretion and potential treatment strategies to increase luminal alkalinity. In the last part of the review, the cellular and organismal mechanisms for acid/base sensing in the intestinal tract are highlighted.

## Historical perspective

Scientists recognized more than a century ago that the gastric mucosa does secrete not just acidic but also alkaline fluids, both of which could be stimulated [125]. The interrelatedness of high gastric acidity and a protective alkaline “*Verdünnungsflüssigkeit*,” an alkaline diluent, was also recognized and its origins were hotly debated at the beginning of the last century [14]. More detailed studies of gastrointestinal bicarbonate transport started in the 1970s, after the development of a technique that combined electrophysiological investigations of epithelia in Ussing chambers with pH–stat microtitration techniques to record the rates of proton or base output into the luminal fluid [46, 47, 62]. Another groundbreaking technique was the intestinal perfusion technique, which permitted the perfusion of a segment of the intestine with defined solutions via separate inflow and outflow catheters, isolated from the other parts of the bowel by inflatable balloons, both in laboratory animals and in man [48]. This technique allowed the formulation of the hypotheses that the human jejunum combines fluid absorption with bicarbonate absorption by a parallel operation of electroneutral Na^+^/H^+^ exchange with Cl^−^/HCO_3_^−^ exchange in the brush border membrane [175]. Regional differences in the ratios of the activities of the two types of exchangers could explain proton secretion in the jejunum, in which the rate of Na^+^/H^+^ exchange exceeds that of Cl^−^/HCO_3_^−^ exchange, and bicarbonate secretion in the ileum, in which the rate of Cl^−^/HCO_3_^−^ exchange exceeds that of rate of Na^+^/H^+^ exchange [10].

A similar technique was used to study the response of the human duodenum upon contact of the mucosa with luminal acid. It was shown that patients who had developed duodenal ulcer disease displayed a reduced HCO_3_^−^ secretory response to a contact of the duodenal mucosa with acid, even after the ulcers had been healed by pharmacological inhibition of gastric acid secretion [74]. Although these results were under debate at a time when it became clear that a very high percentage of patients with duodenal ulcer disease harbored a chronic infection with H*elicobacter pylori* in their antrum [69, 109], it is now clear that the findings were likely due to the gastric metaplasia in the duodenum [16]. This metaplasia is caused by chronic hypersecretion of gastric acid, secondary to the destruction of the complex and finely regulated feedback inhibition of high gastric acidity in the antrum by the *Helicobacter pylori–*induced low-grade antral inflammation [16]. Indeed, the same group showed a decade later that after pH eradication and mucosal healing, the duodenal HCO_3_^−^ secretory response to acid was normalized in former duodenal ulcer patients [63].

In the last century, the study of molecular mechanisms and physiological regulation of intestinal HCO_3_^−^ secretion focused on the upper gastrointestinal (GI) tract, while mechanisms of fluid secretion and absorption were studied in the lower GI tract. Only a few studies addressed the role of an alkaline micromilieu for the restitution of the colonic mucosa after injury [45, 130]. Therefore, it was not realized for a long time that the rates of HCO_3_^−^ output in the colon are far higher than those in the duodenum [12]. The physiological significance of these high colonic HCO_3_^−^ output rates is not limited to Cl^−^ reabsorption from the lumen but also plays an important role in the luminal pH-maintenance during bacterial fermentation, because the generated fermentation products (short-chain fatty acids (SCFA), ammonia/ammonium ions, and carbonic acid/CO_2_) need to be neutralized [11, 163]. Because of the decreasing incidence and better manageability of gastric and duodenal ulcer disease, in parallel to an increased incidence and severity, as well as more treatment resistance, of inflammatory bowel diseases (IBD), the interest has recently shifted and more studies on alkaline output and the involved transport and regulatory mechanisms have been performed in the ileocolon, which will be discussed later. Another area of intense research became the defective HCO_3_^−^ transport in the epithelia of patients with cystic fibrosis [98].

## Classic techniques and recent technological advances for the study of intestinal HCO_3_^−^ transport

More than 10 years ago, a review has carefully described the techniques which were then available to measure cystic fibrosis transmembrane conductance regulator (CFTR)–dependent HCO_3_^−^ transport, which was the most interesting aspect of alkaline output for scientists involved in research related to cystic fibrosis [72]. Another review published at that time addressed the novel techniques that had increased the armamentarium to study HCO_3_^−^ transport in transgenic mice. Major advances have been made in the assessment of surface epithelial function using video-imaging and two-photon techniques to assess the intracellular and juxtamucosal pH in anesthetized rodents and isolated tissues using dye methods, and combine microelectrode techniques, laser Doppler flow, and optical techniques to simultaneously assess blood flow, pH, and mucus layer buildup. In addition, the techniques to determine alkalinization rates of different segments of the murine gastrointestinal tract and of organs that secrete alkaline fluids into the digestive tract have become more sophisticated, in part due to miniaturization of equipment to keep a perfect systemic blood pressure and acid/base control in anesthetized transgenic mice [144].

During the last decade, three novel developments have revolutionized the way how we are studying HCO_3_^−^ transport processes: The first is an ability to preserve the intestinal stem cells that are present in the cryptal region, to generate intestinal epithelium from these stem cells, and to differentiate the epithelium in a segmental organotypic fashion [34, 140, 169]. The second is the elucidation of all components of the “gene scissor” CRISPR-Cas9 prokaryote immune defense system [83] and to optimize its applicability for the use in mammalian cells [31, 84, 107]. The third technical advance is the ability to continuously measure the pH directly at the apical surface of a cell monolayer and thus investigate acid/base flux via the apical membrane (although flux rates cannot be exactly quantitated) [135]. A combination of these techniques may allow a much better delineation of the importance, transport mode, and regulation of the individual HCO_3_^−^ transporters expressed along the crypt-villus axis, particularly in the human GI tract.

## Current understanding of bicarbonate transport in the different segments of the gastrointestinal tract

To the knowledge of the authors, no previously unrecognized HCO_3_^−^ transporters have been described in intestinal tissues in the last decade. Since a number of excellent recent reviews have recently been published that describe the transport mode of individual HCO_3_^−^ transport proteins in detail, we will focus on the physiological significance of individual HCO_3_^−^ transporters, on their interplay with other transporters, and on intervention strategies to improve pathological HCO_3_^−^ transport dysfunction. Because of the multitude of investigations, this review will focus on information obtained in mice and where available in human tissue.

### Basal luminal alkalinization rates in the different intestinal segments

The alkalinization rates of different segments of isolated and chambered murine intestinal mucosa are very different, with particularly low rates in the jejunum and proximal colon and particularly high rates in the cecum and mid-distal colon [89, 196]. The same pattern is observed when the different segments are perfused with an unbuffered electrolyte solution of neutral pH in anesthetized mice. In the distal intestine, the basal alkalinization rates in the different segments correspond well with the expression levels of the luminal Cl^−^/HCO_3_^−^ exchanger Slc26a3 [165] and are only minimally reduced in the absence of CFTR [196]. In the proximal intestine, basal alkalinization rates are 3–fourfold lower than in the mid-distal intestine and are reduced in the absence of CFTR [146, 196], Slc26a3 (DRA) [157, 182], and to a lesser extent of Slc26a6 (PAT-1) [185]. These results are similar in vitro and in vivo, and they only apply for a situation with unbuffered luminal saline or Ringer’s solution.

### Effect of nutrients on basal alkalinization

The situation changes when nutrients are present in the luminal perfusate. The addition of glucose to the luminal bath of chambered small intestine results not only in a strong increase of Na^+^ and fluid absorption but also in a decrease in alkalinization rates in vitro [146], as well as a decrease in the jejunal pH-microclimate in vivo. Among other mechanisms, the intracellular utilization of glucose generates acidic moieties that leave the cell via luminal Na^+^/H^+^ exchangers [33, 102]. The addition of short-chain fatty acids (SCFA), which are important nutrients for the colonocyte, to the colonic mucosa is associated with an increase in HCO_3_^−^ output [7, 176]. The explanations for this phenomenon varied widely, including the postulation of a SCFA/HCO_3_^−^ exchanger in the luminal membrane [178]. We now know that monocarboxylate transporters which are either proton coupled (MCT1) or sodium coupled (SMCT1) are expressed in the apical membranes of colonocytes and that SCFA can also transverse the apical membrane in a non-dissociated form when the luminal pH is low [160]. The cotransport of SCFA with H^+^ via MCT1 (likely the predominant transport protein for SCFA [115]) will result in a removal of protons from the lumen, which is equivalent with an alkalinization. In addition, MCT1-mediated SCFA absorption will acidify the colonocyte, resulting in a stimulation of the apical NHE3 and thus Na^+^ and fluid absorption. However, because SCFA anions are cotransported with protons via MCT1 and MCT4 in the basolateral membrane into the blood stream, the apical proton recycling via NHE3 does not match the rate of proton disappearance from the lumen, and a luminal alkalinization is observed. In line with the predominant site of microbiome-mediated SCFA production in the proximal colon, both MCT1 and NHE3 are strongly expressed in the mucosa of the proximal colon, both in mice and humans, whereas Slc26a3 is weakly expressed [145, 160]. This results in low alkalinization rates in the presence of luminal saline but high rates in the presence of luminal SCFA anions [178]. These are just two examples how the presence of nutrients in the lumen may completely change the alkalinization rates both in vivo and in vitro.

### Secretagogue-stimulated intestinal HCO_3_^−^ secretion

In the intestine, the CFTR anion channel remains the most important, if not the only, conductive pathway for HCO_3_^−^ exit under agonist-stimulated conditions. If heterologously expressed, the HCO_3_^−^ conductivity of CFTR is only approximately one-fifth of that for Cl^−^ under maximal electrochemical gradients [126, 168]. Recent data have shown how the CFTR channel may switch from a predominantly Cl^−^-conductive to an HCO_3_^−^-conductive channel through the activation of WNK (with-no-lysine) kinase and the phosphorylation of the downstream kinases SPAK and OSR1 in pancreatic ducts cells [123]. WNK signaling is activated when the intracellular Cl^−^ concentration falls to low levels [91]. In the distal pancreatic ducts of guinea pigs (and possibly humans as well), this is a likely scenario during secretin-stimulated pancreatic juice secretion, because the ductal cells express low levels of basolateral NKCC1, which is a major Cl^−^ importer during stimulated anion secretion [200]. In murine duodenum, phosphorylated SPAK and OSR1 are very abundant [196], which may be one reason why, even without exogenous stimulation, the percentage of HCO_3_^−^ secretion that is dependent on luminal Cl^−^ (and therefore presumably mediated by Cl^−^/HCO_3_^−^ exchange) is much lower than in the murine colon, and the percentage that is dependent on CFTR expression is high [155]. A capsule pH-metry in CF patients displayed a strong difference in the neutralization capacity of the proximal but not the distal intestine [53]. A CFTR-targeted therapy significantly improved the acid-neutralizing capacity of the proximal intestine [54]. The regulation of the CFTR channel by hormonal, neural, and luminal agonists and endogenous inhibitors is complex and not the topic of this review.

Another mode of CFTR-dependent, agonist-stimulated HCO_3_^−^ secretion is a functional interaction between the agonist-activated CFTR Cl^−^ conductance and an apical Cl^−^/HCO_3_^−^ exchanger, which recycles the luminal Cl^−^ in exchange for HCO_3_^−^ [44, 71, 101]. For this coupling mechanism to become operative, the two transporters need to be coexpressed in the apical membrane of the same cell type, a requirement for apical Cl^−^ recycling, rather than rapid import of Cl^−^ via the basolateral membrane needs to exist, and an ample supply of HCO_3_^−^ to the cell needs to be present. All these requirements appear to be met in the proximal pancreatic duct cells, which coexpress CFTR and SLC26a6 in the apical membrane [76, 186] and have high expression levels for the basolateral Na^+^/HCO_3_^−^ cotransporter NBCe1 [201] and low expression of the basolateral AE2, which would export HCO_3_^−^ in exchange for Cl^−^ and therefore lower the availability of intracellular HCO_3_^−^ for apical exchange [133].

In the intestine, the situation is different, because the expression of CFTR and NKCC1 is strongly crypt predominant, and that of NBCe1 and the apical SLC26 anion exchangers is villus/surface predominant [78, 79, 152, 185]. An area of overlapping expression along the crypt/villus (surface) axis is necessary for CFTR-dependent HCO_3_^−^ secretion via apical Cl^−^ recycling. Because of insecurities with antibody specificity, technical issues, and species differences, the literature is equivocal regarding the expression pattern and the relevance for agonist-induced HCO_3_^−^ secretion for either of the three SLC26 members expressed in the duodenum, namely, SLC26A3 (DRA), SLC26A6 (PAT-1), and SLC26A9 as well as of CFTR. While most publications report a crucial role for a functional CFTR in cAMP-, cGMP-, and Ca^2+^-dependent stimulation of duodenal HCO_3_^−^ secretion in vitro [30, 64, 146] and in vivo [64, 65, 155], some reports found only a minor or minimal role [139, 149]. The reason for these discrepancies might be due to technical issues. There is indeed an agonist-mediated pathway of luminal alkalinization that is independent of CFTR expression and operates in murine as well as human intestine: The agonist-induced inhibition of apical proton extrusion mechanisms such as the Na^+^/H^+^ exchanger isoform NHE3, which is highly expressed in the brush border membrane of most intestinal segments except the distal colon, or of the distally expressed colonic H^+^/K^+^ ATPase will result in a CFTR-independent increase in luminal alkalinization in the presence of a highly expressed and cAMP-insensitive SLC26A3 [145, 166]. It has also been suggested that SLC26A3 may traffic to the brush border membrane in a cAMP-dependent but CFTR-independent fashion [172].

### Role of SLC26A6 in intestinal HCO_3_^−^ secretion

Slc26a6 is strongly expressed in the proximal intestinal tract, with a gradient towards much lower expression in the colon [184]. If the expression levels are compared in the same sample of laser-dissected mucosal cells, mRNA expression of Slc26a6 is somewhat higher in the adult murine villous area from the duodenum than that of SLC26a3 expression, and both transporters are expressed in a villous-predominant fashion [103]. In the suckling murine intestinal mucosa, Slc26a6 mRNA expression levels were lower than for Slc26a3 in all small intestinal segments [205]. Slc26a6 is discussed as an electrogenic transporter, exporting two HCO_3_^−^ ions and importing one Cl^−^ ion [75, 82, 119, 131]. Other reports find evidence for an electroneutral Cl^−^/HCO_3_^−^ exchange [4, 26, 183].

When *slc26a6*^−/−^ and WT chambered duodenal mucosae were compared in Ussing chambers and the HCO_3_^−^ secretion rate into a CO_2_/HCO_3_^−^ free luminal electrolyte solution was measured (which offers a perfect driving force for a 2 HCO_3_^−^_out_/1 Cl^−^_in_ exchange), a contribution of Slc26a6 for luminal alkalinization was observed, but the relative importance was less than expected, since the majority of basal and cAMP-dependent HCO_3_^−^ secretion was preserved in the absence of Slc26a6 expression [173, 185]. Interestingly, Slc26a6 is involved in fluid absorption in isolated jejunal mucosa [148] and in anesthetized mice [156, 195]. In addition, Slc26a6 was shown to mediate HCO_3_^−^ import during PEPT-1 mediated enterocyte acidification [153]. Another study demonstrated that the role Slc26a6 in basal and stimulated duodenal HCO_3_^−^ secretion was dependent on the systemic acid/base status of the anesthetized mice [155]. Therefore, while the role of Slc26a6 in small intestinal oxalate secretion is well established [49, 50, 94], its exact physiological function as an intestinal HCO_3_^−^ transporter is less clear.

### Role of SLC26A3 in intestinal HCO_3_^−^ transport

SLC26A3 was one of the first members of the gene family whose molecular sequence was identified during a screening of a subtraction cDNA library constructed from colonic normal epithelium and adenomatous and cancer tissue and named downregulated in adenoma (DRA) [142]. A group of Finnish geneticists had narrowed the chromosomal location of the presumed gene that was defective in the rare hereditary chloride-losing diarrhea (CLD) to a narrow locus [90], and this enabled them to rapidly identify the *DRA* gene locus as the locus for all mutations know so far known in CLD [66]. This quickly established its potential role as the gene encoding for a luminal Cl^−^/HCO_3_^−^ exchanger, hypothesized to be defective in CLD [174]. However, it took several more years until the transporter was functionally identified to be a *bona fide* Cl^−^/HCO_3_^−^ transporter [100, 113, 114]. Its stoichiometry is still debated: Some reports suggest an electrogenic 2 Cl^−^_i_/1 HCO_3_^−^_o_ exchange [151], while others suggest an electroneutral exchange [5, 100].

A *slc26a3*^*−/−*^ mouse model was established, which recapitulated many features of CLD in humans [143]. Massive hyperaldosteronism and its sequelae were reported, namely, the strong upregulation of the Na^+^/H^+^ exchanger NHE3 and the epithelial Na^+^ channel ENaC in the colon of *slc26a3*^−/−^ mice. However, this rescue mechanism may be less effective than originally thought, because the mid-distal colon of these mice did not absorb fluid [198]. The likely reason is that the acidic luminal and stool pH of these mice [92] inhibits apical Na^+^_i_/H^+^_o_ exchange due to the high affinity of protons to the external binding site of the transporter. When the luminal pH is buffered to 7.4, the *slc26a3*^*−*/−^ mid-distal colon is able to absorb fluid at approx. 50% of the rate seen in WT littermates ([166] and unpublished observations).

The *slc26a3*^−/−^ mice have extremely low colonic luminal alkalinization rates [198] and a very acidic surface pH and develop a mild distal colitis over time [92]. This feature is interesting because CLD patients also have a high incidence of inflammatory bowel disease [117, 189]. A search for potential underlying mechanisms included a lack of an adherent mucus layer [198] as well as alterations in mucus formation and release [92], a dysbiotic microbiome [92, 93], a reciprocal cellular expression and action of TNFα and SLC26A3 [41], and disturbances of tight junction regulation [97, 207]. An interesting recent finding was a very strong upregulation of the expression of several classes of antimicrobial peptides in the *slc26a3*^−/−^ colonic mucosa, which may in part explain the surprisingly mild inflammation in the *slc26a3*^−/−^ colon, which occurs late in life, despite mucus abnormalities and dysbiosis from shortly after birth [92, 93]. However, a recent investigation reported a proinflammatory effect of an overproduction of Reg3, one of the antimicrobial proteins strongly upregulated in *slc26a3*^−/−^ colonic mucosa [93], via a reduction in *Enterococcus species* that were protective in C57B/6 mice against DSS-induced colonic inflammation [80]. *Enterococcus species* were not abundant, and not different between genotypes, in the microbiota in our *slc26a3*^−/−^ and WT mouse cohort (also C57B/6 background). This demonstrates how mouse house-associated factors may impact the outcome of research related to the highly complex pathophysiology of intestinal inflammation.

### Effect of basolateral HCO_3_^−^ transporting mechanisms on basal and stimulated alkalinization rates

**Fig. 1:**
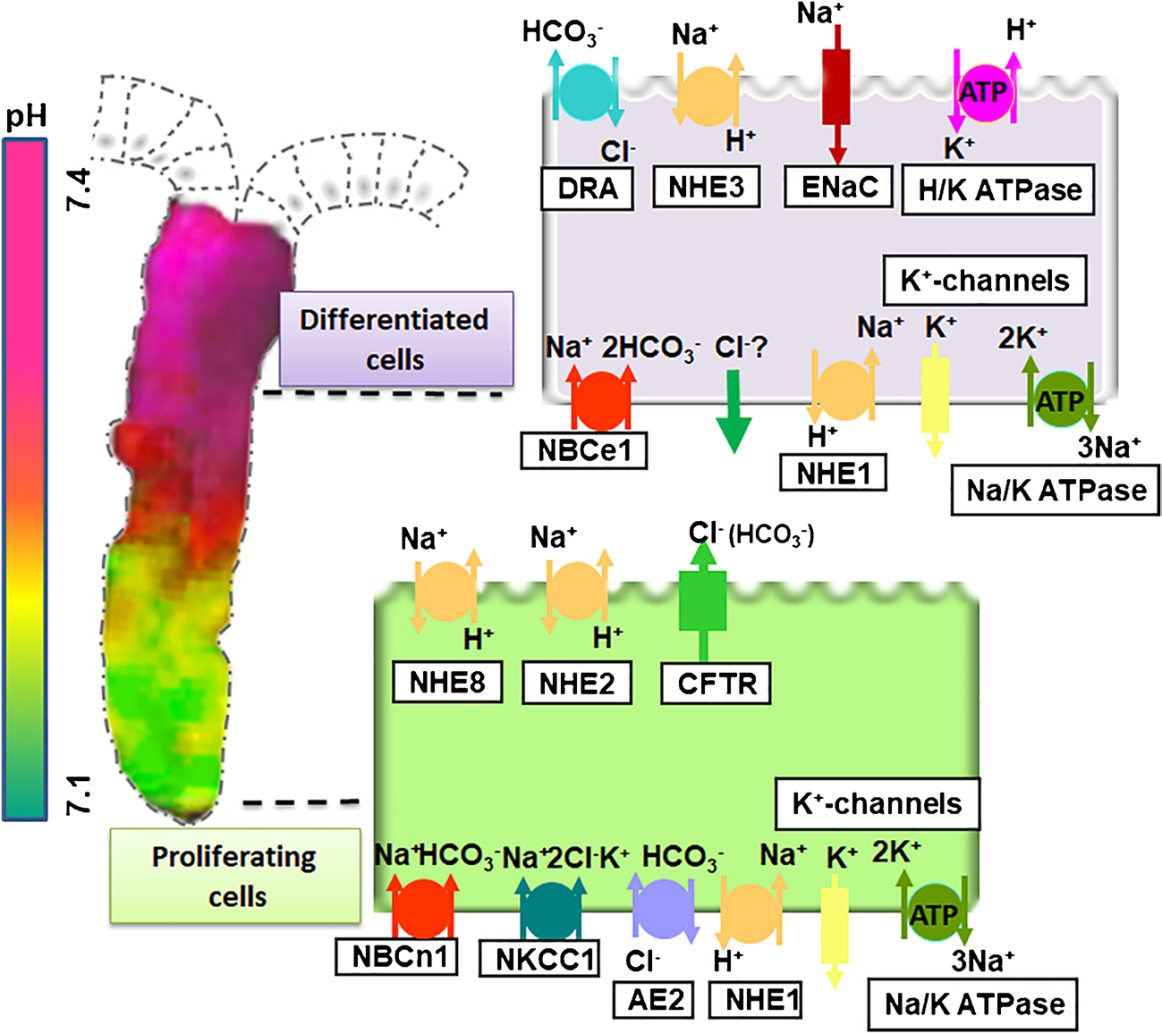
Differential expression of the acid/base transporters along the human colonic cryptal axis. As deduced from studies in proliferating and differentiated human colonic enteroids, in situ hybridization, immunohistochemical staining, and fluorometric pH_i_-measurements, the acid/base transporters are differentially expressed along the cryptal axis. The ion transport machinery for electrogenic anion secretion, with the lead components CFTR and NKCC1, is coexpressed with AE2, NBCn1, and TMEM16a in the highly proliferative lower cryptal region and strongly downregulated during enterocyte differentiation. In contrast, the acid/base transporters SLC26a3, NHE3, and NBCe1 and the colonic H^+^/K^+^ ATPase, together with the ENaC subunits, are upregulated in the differentiated absorptive enterocytes. The intracellular pH is more acidic in the cryptal region than in the surface enterocytes. The full names of the abbreviations can be found in the manuscript

Early studies in chambered amphibian duodenum noticed that luminal alkalinization required the presence of physiological concentrations of Na^+^ and HCO_3_^−^ in the serosal bath [154]. In the rabbit duodenum, neither the specific inhibition of the basolateral Na^+^/H^+^ exchanger NHE1 nor of a Na^+^/HCO_3_^−^ cotransporter had a significant effect on basal or stimulated HCO_3_^−^ secretion, but the combined inhibition of both pathways effectively reduced the alkalinization rate [77]. The contribution of the electroneutral Na^+^/HCO_3_^−^ cotransporter NBCn1 (SLC4A7) to duodenal HCO_3_^−^ secretion was confirmed by studying cAMP-dependent duodenal HCO_3_^−^ secretion in vitro [24] as well as luminal acid–induced HCO_3_^−^ secretory response in vivo [158] in *slc4a7*^−/−^ mice. NBCn1 is relatively DIDS-insensitive [27, 132] and was found to be expressed in the murine and human duodenum [13, 32]. To study an involvement of the electrogenic NBCe1 in intestinal HCO_3_^−^ secretion proved very difficult, because the mice die during weaning [52]. Although they display reduced HCO_3_^−^ import into enterocytes in response to nutrient- or electrolyte transport–induced acid loads and a decreased basal alkalinization rate in the small, but not the large intestine, as well as a reduced anion and fluid secretory response to cAMP stimulation, the cAMP-induced HCO_3_^−^ secretory response was not different between the small intestinal and colonic mucosa of the suckling *slc4a4*^−/−^ and WT littermates [205]. It was noticed in that study that the HCO_3_^−^ uptake rates into the surface colonocytes, but not into colonocytes in the cryptal base, were significantly reduced in *slc4a4*^−/−^ compared to WT mice, whereas the opposite was found for surface and cryptal base colonocytes of *slc4a7*^−/−^ mice. This suggested that there may a differential expression of the two NBCs along the cryptal axis and therefore a differential effect of their deletion on basal vs cAMP-induced HCO_3_^−^ secretion. A recent study in human colonic organoids confirmed the strong expression of the electroneutral NBCn1, together with other components of the anion secretory machinery, namely, CFTR, NKCC1, TMEM16a, and AE2, in the non-differentiated, proliferative enteroids, with a strong downregulation of their expression during differentiation [136]. In contrast, the electrogenic NBCe1 was upregulated during differentiation. This also makes sense functionally, because a cAMP-induced opening of basolateral K^+^ channels, which increases the electrochemical gradient for apical anion secretion, at the same time, reduces the electrochemical gradient for NBCe1-mediated HCO_3_^−^ import, while it does not affect that for NBCn1. Thus, NBCn1 may play an important role for agonist-induced CFTR-dependent HCO_3_^−^ secretion and may augment the NKCC1-independent AE2-dependent Cl^−^ import into colonic crypts [141]. In contrast, the cellular acidification mediated by H^+^-coupled dipeptide, by H^+^-coupled short-chain fatty acid anion import, or by apical Cl^−^/HCO_3_^−^ exchange in the surface cells either depolarizes the membrane potential or does not affect it, permitting electrogenic 1 Na^+^/2 HCO_3_^−^ import (Fig. 1).

## Strategies to influence luminal alkalinity in patients with defects in intestinal HCO_3_^−^ transport

### Cystic fibrosis

**Fig. 2:**
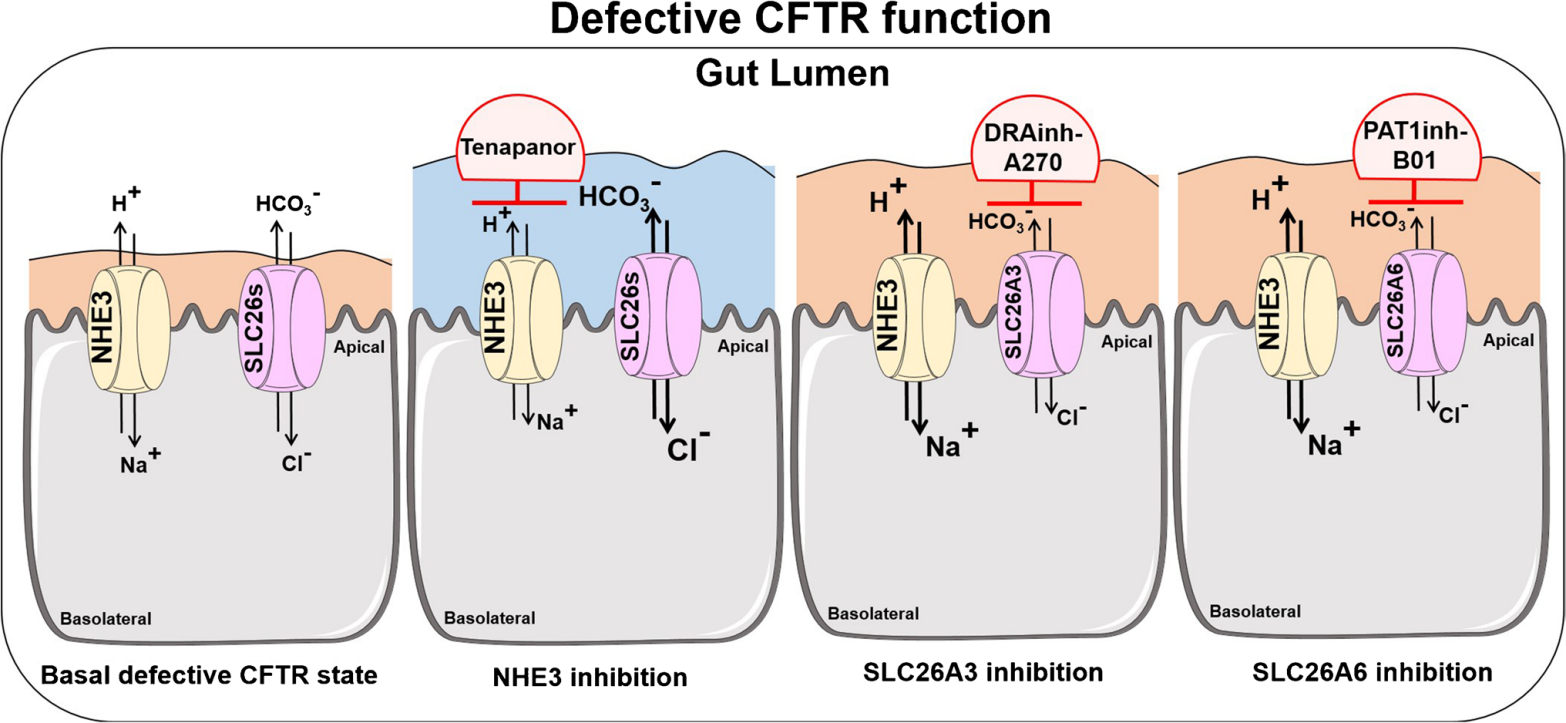
Schematic diagram of pharmacological strategies to increase the luminal fluidity in the CFTR-deficient intestine. In the left panel, the situation in the villous/surface enterocytes of the CFTR-deficient intestine is depicted. The ongoing absorptive activity of the absorptive enterocytes, together with the lack of fluid/alkaline secretion from the CFTR-expressing cryptal region, results in an acidic, dehydrated gut lumen. Left-middle panel: Oral inhibition of the apical Na/H exchanger isoform NHE3 with the intestine-specific selective NHE3 inhibitor tenapanor results in an increase in luminal fluidity and alkalinity in the intestine of CFTR null or F508del mutant mice [141, 149]. Middle-right and right panel: The inhibitors for SLC26A3 [112] and SLCA6 [108] are also able to inhibit fluid absorption and may be beneficial to alleviate constipation and obstructive episodes in patients with CF. They are not expected to increase the luminal alkalinity, however. The full names for the abbreviations can be found in the manuscript

Patients with cystic fibrosis have traditionally been treated with laxatives to prevent recurrent obstructive episodes and with agents that inhibit acid secretion to mitigate reflux symptoms and optimize digestion. These suboptimal strategies to improve CF-associated intestinal disease are now being successfully replaced by CFTR targeted therapy which improves not only the pulmonary but also the intestinal symptoms [54, 88, 112]. Preliminary results suggest that this improvement is not reached via an alteration of the dysbiotic gut microbiota seen in patients with CF [108]. Recent studies in CFTR null mice aimed to explore strategies to increase the alkalinity and fluidity in an intestine that expressed no functional CFTR protein at all and therefore is not a candidate for CFTR-targeted therapy. Indeed, several FDA-approved agents were identified that increased the luminal alkalinity and reduced small and in part also large intestinal fluid absorptive rate [166, 167] or reduced the CF-associated delayed small intestinal transit time [111]. Other experimental drugs also carry the potential to increase gut fluidity, but have not yet been tested in CFTR-deficient mice [28, 59]. Two agents, one of them a TMEM16a/SLC26A3 inhibitor [181] and the other one an intestine-specific, selective, and FDA-approved NHE3 inhibitor [167], were able to significantly reduce the frequency of intestinal obstructions in CFTR null mice. Both agents also reduced the mucus impactions in the intestine The latter study demonstrated a reversal of several of the “CF gut” features within the short 3-week period of the application period, including a decrease in cryptal hyperproliferation, mucus accumulation, and mucosal mast cell number. Figure 2 schematically explains the mode of action of NHE3 and SLC26A3/A6 inhibitors may increase luminal fluidity in the CF gut. Only NHE3 inhibition will result in both an increase in fluidity and alkalinity in the CF gut. These studies raise hope that pharmacological therapies will soon be available to ameliorate the intestinal symptoms in all patients with cystic fibrosis.

### Congenital chloride diarrhea CCD (chloride-losing diarrhea, CLD)

Early intestinal perfusion studies in CLD patients localized the defective transport to the ileocolon [174]. Therefore, compensatory mechanisms, such as the expression of SLC26a6, passive Cl^−^ absorption, or the predominant role of nutrient-coupled electrolyte absorptive mechanisms, may mitigate the effects of a defective SLC26a3 in the small intestine of patients with CLD. In the large intestine, alternative fluid and electrolyte uptake systems are available, but their efficacy may be compromised by the low luminal pH [92, 93, 198] and by the dysbiosis with a strong reduction of SCFA-producing bacteria [93]. Indeed, the luminal application of SCFA in the concentrations that are present in the healthy colon increased the alkaline output into the luminal solution in isolated chambered *slc26a3*^−/−^ mucosa [60], and it resulted in a strong increase in fluid absorptive rates in the *slc26a3*^−/−^ cecum and colon in anesthetized mice (Tan, Qinghai. 2021, unpublished observations). However, an oral application of butyrate salt or of tributyrin did not significantly increase the cecal and colonic SCFA levels and did not reverse the diarrhea in *slc26a3*^−/−^ mice (Ye, Zhenghao and Kini, Archana, unpublished observations), demonstrating that even high doses of butyrate, taken orally, are completely absorbed in the small intestine of mice. Consistent with these observations, the oral application of butyrate to patients did not result in an improvement of the CLD biomarkers [190].

However, one pediatric center was able to apply surprisingly high doses of oral butyrate to CLD children and normalize their stool and serum parameters [18, 40]. Since oral butyrate salts firstly smell very bad even in a microencapsulated form, need to be applied in high doses, and are absorbed in the small intestine, the challenge will be to deliver the high millimolar concentrations required for effective SCFA-dependent fluid absorption to the colon. Possibly, a strategy will be developed to replenish the missing colonic microbiota and thereby enhance local production of SCFA in the colon of CLD patients.

### Inflammatory bowel disease

Many studies have been conducted to explore the electrolyte transport abnormalities in the inflamed mucosa of IBD patients of animal models of intestinal inflammation, summarized in reviews that have been published in the last decades [6, 106, 110, 147, 204]. One prominent feature of IBD is a strong decrease in the expression of SCL26a3 in the inflamed colonic epithelium, resulting not only in reduced electrolyte and fluid absorption but also in a reduction in luminal alkalinization [197]. However, the paracellular leakage of HCO_3_^−^ through the tight junctional pathway with a loss of cation selectivity may offset the reduced SLC26A3-mediated luminal alkalinization, in particular in the ileum and proximal colon, where SLC26A3 expression is much lower than in the mid-distal colon [86]. This may be a reason why pH capsule investigations of patients with IBD gave mixed results [118, 127, 202]. Polymorphisms in SLC26A3 have been associated with either an increased incidence of IBD [9, 51, 150] or have been associated with response to therapy [8, 17, 188]. Therefore, intense research has been ongoing to elucidate the potential role of SLC26A3 in mucosal protection, as mentioned above, and in the maintenance of barrier properties [19, 81]. The hope is to somehow restore Slc26a3 function in IBD [58, 128, 199]. However, recent evidence suggests that the downregulation of SLC26A3 observed in the inflamed mucosa of IBD patients is just one component of a dysregulated differentiation process in the inflamed mucosa which prevents the full differentiation of absorptive enterocytes [116]. Therefore, the only effective strategy to improve the expression and function of the absorptive ion transporters may be an effective anti-inflammatory treatment.

## Acid/base sensing in the intestine

As already described in the previous chapters, luminal pH has to be tightly controlled to provide adequate defense of the intestinal epithelium against luminal acid and to maintain homeostasis. Therefore, the concentration of acid/base equivalents is tightly monitored by a sophisticated network of proton- and bicarbonate-sensing proteins, which are situated in epithelial cells and sensory neurons along the gastrointestinal tract. These acid/base-sensing proteins comprise acid-sensitive ion channels (ASICs) and transient receptor potential cation channels (TRPs) [67, 124, 164, 206]. But also, other acid/base-sensitive proteins like soluble adenylyl cyclase (sAC), two-pore domain potassium (TASK) channels, H^+^-sensing G-protein-coupled receptors (GPCRs), P2X receptors, and inward rectifier potassium (Kir) channels could play a role in gastrointestinal pH sensing [55, 57, 68, 70]. Besides pH-sensing proteins, efficient acid/base sensing also requires acid/base transporters and carbonic anhydrases (CAs) [3].

### Luminal acid/base sensing requires catalytic function of carbonic anhydrases

CAs catalyze the reversible hydration of CO_2_ to HCO_3_^−^ and H^+^. Due to the fast interconversion, CO_2_, HCO_3_^−^, and H^+^ form a “trinity,” which allows both H^+^ and HCO_3_^−^ sensors to monitor changes in pH, bicarbonate concentration, and CO_2_ partial pressure at the same time.

**Fig. 3:**
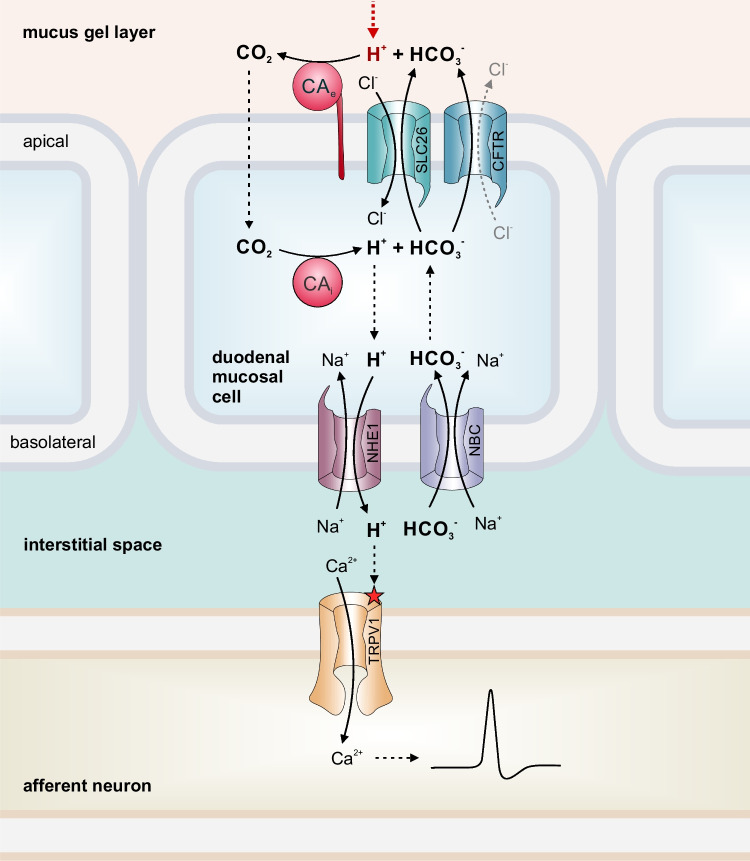
Acid sensing by afferent neurons in the duodenum is mediated by the collaboration between carbonic anhydrases, acid/base transporters, and acid receptors. Protons, which enter the mucus gel layer, are neutralized by HCO_3_^−^, which is exported from the duodenal mucosal cell via Cl^−^/HCO_3_^−^ exchangers and CFTR. The reaction of H^+^ and HCO_3_^−^ to CO_2_ is catalyzed by extracellular CA. CO_2_ diffuses into the cell, where it is again hydrated by intracellular CAs. While HCO_3_^−^ is again exported on the apical side to neutralize acid, H^+^ is exported from the cell on the basolateral side via NHE1. H^+^ can then activate H^+^ receptors like TRPV1 on the surface of neurons of afferent nerves, to induce neuronal activity. Figure modified from [2, 68]

A good example of how CAs, acid/base transporters, and acid receptors work together to monitor luminal pH is acid sensing by afferent neurons in the duodenum, as first proposed by Akiba and colleagues [2, 3] (Fig. 3): Luminal pH in the duodenum can rapidly change between 2 and 7, due to bursts of acid from the stomach. Therefore, the duodenal mucosa has to be able to rapidly adjust its defense mechanism against luminal acid. The acid is neutralized by HCO_3_^−^, which is secreted from duodenal epithelial cells by bicarbonate transporters from the SLC26 family and the cystic fibrosis transmembrane conductance regulator (CFTR). In the mucous layer, H^+^ and HCO_3_^−^ react to CO_2_, catalyzed by extracellular CAs, which are situated at the apical site of the membrane. The CO_2_ diffuses from the mucus layer into the cell, where it is hydrated to H^+^ and HCO_3_^−^, catalyzed by cytosolic CA. While HCO_3_^−^ is again secreted at the apical site to support neutralization of luminal acid, H^+^ is exported into the interstitial space via Na^+^/H^+^ exchanger 1 (NHE1) at the basolateral site of the epithelial cell. The H^+^ activates proton receptors, situated on adjacent neurons of afferent nerves, which in turn stimulate secretion of HCO_3_^−^ and mucus to protect the mucosa from further acidosis. Through this cascade, luminal pH can be precisely monitored by neuronal acid sensors, even if these nerve fibers have no direct access to the lumen [2, 3]. This hypothesis has been tested by assessing the intracellular pH (pH_i_) of the duodenal villi during acid exposure in vivo by two-photon microscopy in mice that were deficient for the major intracellular CA isoform in the duodenocytes, namely, CAII, and their WT littermates [161]. The authors found that in WT mice, a 5-min exposure of the duodenal mucosa to a pH of 2.2 resulted in strong decrease of the duodenocyte pH_i_ in the villus tip and mid-villus region, which was strongly blunted in the absence of CAII. In parallel, the acid-induced bicarbonate secretory response was virtually ablated in the CAII-deficient duodenum, but only mildly reduced during perfusion with a membrane-impermeable CA inhibitor. Thus, the conduction of an “acid” signal through the duodenocyte to the acid-sensing receptors on neuronal structures requires both luminal and intracellular CAs. However, the CAII was not essential for the HCO_3_^−^ stimulatory response of the epithelium to secretagogues such as forskolin or the stable PGE2 analogue 16,16,-dimethyl PGE2.

### Role of acid-sensing ion channels in gastrointestinal acid/base sensing

Acid sensing in afferent nerve fibers of the gastrointestinal tract is mainly mediated by the transient receptor potential cation channel subfamily V member 1 (TRPV1), also termed capsaicin receptor or vanilloid receptor 1. TRPV1 belongs to the transient receptor potential (TRP) channels. TRPs comprise six families, five of which (TRPV, TRPM, TRPA, TRPP, TRPC) are relevant for chemo-, thermo-, or mechanosensation [29]. Out of these five subfamilies, only TRPV1 has been attributed a function in gastrointestinal acid sensing. TRPV1 is a non-selective cation channel with a high Ca^2+^ permeability, but can also function as proton channel at low pH [61, 68]. The channel is opened at acidic pH values below 6; however, mild acidity can sensitize TRPV1 to other stimuli like heat [68, 85, 170].

Acid-induced secretion of bicarbonate in the duodenum can also be mediated by acid-sensitive ion channels (ASICs), which are expressed in intestinal epithelial cells [42, 208]. ASICs are proton-gated cation channels, which are activated by protonation of the extracellular loop and therefore serve as detectors for extracellular acidification [96, 180, 193]. ASICs have a high permeability for Na^+^ ions; however, some isoforms also show substantial conductance for other cations like Ca^2+^ or K^+^ [68, 208]. In mammals, ASICs are encoded by three different genes (ACCN1, ACCN2, and ACCN3). These three genes produce the five protein isoforms ASIC1a, ASIC1b, ASIC2b, and ASIC3 by alternative splicing, which can form homo- and heterodimers [68]. ASICs have been attributed various physiological and pathophysiological functions, including gastrointestinal mechanoreception [121], learning and memory [191, 203], olfaction [177], pain sensation and fear [38, 39, 192], heart failure [56], and neuronal disorders like epilepsy, mood disorders, and Alzheimer’8520/s disease [162]. Furthermore, ASICs play a role in gastrointestinal pain, gastroesophageal reflux disease, and gastric cancer, where expression of ASIC1a correlates with cancer progression and formation of metastasis [25, 208]. In the duodenum, ASIC1a is thought to be involved in the regulation of bicarbonate secretion [42]: By performing measurements in mouse duodenal epithelial cells and the human intestinal epithelial cell line HT29, the authors could show that extracellular acidification induces an increase in intracellular Ca^2+^ concentration and secretion of HCO_3_^−^. Both Ca^2+^ increase and HCO_3_^−^ secretion were inhibited by inhibition of ASICs with amiloride. From this, it was concluded that ASICs, expressed in duodenal epithelial cells, can induce duodenal HCO_3_^−^ secretion via a Ca^2+^-dependent pathway [42].

### Proton-sensing G-protein coupled receptors in the gastrointestinal tract and their role in IBD

Proton-sensing G-protein coupled receptors (GPRs) serve as detectors for extracellular acidification in almost every tissue. H^+^-sensitive GPRs comprise of the four members GPR4, GPR65 (TDAG8), GPR68 (OGR1), and GPR132 (G2A) [104, 159]. GPR132, however, displays only weak proton-sensitivity and was not found to have pH-sensitive functions [129]. GPR4 and GPR68 are ubiquitously expressed, including the GI tract, while expression of GPR65 is restricted to lymphoid tissue [159]. GPR4 and GPR65 have both been attributed functions in inflammatory bowel disease (IBD).

Inflammation promotes glycolytic energy production, which is attributed to the increased demand for energy by infiltrating immune cells and to local hypoxia of the mucosal tissue, which results in increased formation of lactate and protons [37, 95, 122]. The acidification activates GPR4 and GPR65 with severe consequences. Activation of GPR4 results in increased expression of various inflammatory genes, including different chemokines and cytokines, adhesion molecules, and COX-2. Furthermore, it activated stress-response genes, such as ATF3 and CHOP, and increased cell adhesion though the cAMP/Epac pathway [23, 43]. Thereby, GPR4 can exacerbate inflammation, which results in a positive feedback loop that again activates GPR4 and drives further inflammation [187]. Indeed, it was shown that absence of GPR4 or pharmacological inhibition attenuates colitis in an IBD mouse model, including less severe inflammation [137, 138]. It was therefore concluded that GPR4 might serve as a potential drug target for the treatment of IBD.

GPR68 does also play a role in IBD, mainly by regulation of the intestinal barrier function [35, 36]. GPR68 is coupled via the G-protein G_q11_ and activates an intracellular signaling cascade with phospholipase C (PLCβ), inositol phosphate 3 (IP3), elevation of Ca^2+^, and extracellular signal-regulated kinase (ERK) [104], but it was also shown to couple via Gα12/13 and the Rho pathway [36].

GPR68 was found to be increased in the mucosa of patients with IBD and inflamed segments showed higher abundance of GPR68 than uninflamed mucosa [37]. Short-term expression of GPR68 is induced by the proinflammatory cytokine TNF, which was also shown to function as a major mediator for IBD-associated inflammation. GPR68 is expressed in endothelial cells, macrophages, granulocytes, and fibroblasts. In fibroblasts, activation of GPR68 by extraocular acidification induced formation of inositol phosphate, Rho activation, and formation of F-actin and stress fibers and does therefore increases epithelial barrier function [36]. In IBD patients, expression of GPR68 positively correlates with the expression of the profibrotic genes *Vim*, *Col3a1*, *Tgfb1*, and *Ctgf* and cellular deposition of collagen [73]. In the CaCo-2 cell line, an acidic pH shift from 7.8 to 6.6 improved barrier function and stimulated the reorganization of F-actin and formation of stress fibers. Furthermore, acidic pH inhibited proliferation and cell migration within a wound healing assay [35]. It was also shown that GPR68 deficiency protects from inflammation in an IL-10 knockout mouse model of IBD [35]. GPR68 also contributed a role in the regulation of ER stress via the IRE1α-JNK pathway, as well as blockage of late stage autophagy [105].

IBD is usually associated with local acidification which can result in severe alterations in epithelial barrier function. Therefore, it would seem that strengthening of intestinal barrier function should be beneficial. However, cell proliferation and migration are required to regain homeostasis. Therefore, chronic activation of GPR68 could exacerbate tissue damage in IBD [36].

### Soluble adenylate cyclase is a bicarbonate sensor in the gastrointestinal tract

A *bona fide* bicarbonate sensor, which is also expressed in the gastrointestinal tract, is soluble adenylyl cyclase (sAC). Adenylyl clyclases catalyze the conversion of ATP to AMP, which functions as second messenger, either by promoting protein phosphorylation via cAMP-dependent protein kinase or by direct activation of cAMP-regulated proteins [134]. Mammals express ten isoforms of adenylyl clyclases. However, out of these ten isoforms, nine are membrane-bound and activated by the G protein α subunit; only the sAC isoform is directly activated by binding of HCO_3_^−^ to the enzyme [55, 87, 120, 179]. Human sAC has an EC_50_ between 11 and 12 mM for HCO_3_^−^, which is close to physiological HCO_3_^−^ concentration and enables sAC to detect already small changes in intracellular HCO_3_^−^ concentration [22, 87]. Therefore, sAC can serve as a key regulator for acid/base-depending cell signaling in various intracellular compartments, including cytosolic microdomains and organelles like mitochondria and the nucleus [1, 209]. A recent study could demonstrate that sAC can regulate the cytosolic NADH/NAD^+^ redox state and is therefore involved in the bioenergetic switch between glycolysis and oxidative phosphorylation [21]. sAC was first found in the testis [15] but is ubiquitously expressed in mammalian tissue, including the intestine [67]. Intestinal HCO_3_^−^ sensing by sAC, however, has been primarily studied in teleost and cartilage fish: Marine fish, which live in a hyperosmotic environment, absorb water through their intestine to compensate for dehydration due to water loss across the gills. Water absorption is driven by absorption of Na^+^ and Cl^−^ via Na–K-2Cl-Cotransporters (NKCC) and Cl^−^/HCO_3_^−^ exchangers in the gastrointestinal tract. Water absorption, however, also requires the removal of divalent cations like Ca^2+^ and Mg^2+^ to further decrease osmotic pressure. Removal of Ca^2+^ and Mg^2+^ is facilitated by HCO_3_^−^, which is secreted in exchange for Cl^−^ via an anion exchanger of the SLC26A6 family on the apical site of intestinal cells [99]. Additionally, HCO_3_^−^ can be imported into the epithelial cell from the apical side via the Na^+^/HCO_3_^−^ cotransporter NBCe1 [99] or luminal CO_2_, which diffuses back into the cell and is converted to HCO_3_^−^ and H^+^ by intracellular carbonic anhydrase in the subapical region of the cytosol [171]. This results in extremely high luminal HCO_3_^−^ concentrations of more than 100 mM. The resulting alkaline conditions induce precipitation of Mg^2+^ and Ca^2+^ ions as MgCO_3_ and CaCO_3_ and reduce osmotic pressure to facilitate water absorption [171, 194]. Furthermore, the HCO_3_^−^ activates sAC. Activated sAC activates the NKCC and triggers intestinal NaCl absorption [20, 194]. By this mechanism, sAC can locally modulate bicarbonate secretion and water absorption in the fish intestine [20, 194].

## Outlook

Many of the reviewed publications have been performed in genetically altered mice. However, this has recently changed, with new studies focusing on HCO_3_^−^ transport and the regulation of enterocyte pH in human intestinal two- or three-dimensional organoids. Although the in vitro differentiation of intestinal organoids still needs refinement, it is anticipated that this technique, in combination with genetic manipulations and novel optical approaches to study acid/base transport in cultured epithelia, will greatly enhance our understanding of the regulation and interplay of the different ion transport proteins and pH sensors along the crypt-villus/surface axes of the different intestinal segments.

## Data Availability

No datasets were generated or analyzed during the current study.

## Abbreviations

AE2: Anion exchanger isoform 2
ASIC: Acid-sensitive ion channel
CA: Carbonic anhydrase
CF: Cystic fibrosis
CFTR: Cystic fibrosis transmembrane conductance regulator
CCD: Congenital chloride diarrhea
CLD: Chloride-losing diarrhea
DIDS: 4,4′-Diisothiocyano-2,2′-stilbenedisulfonic acid
DRA: Downregulated in adenoma (SLC26A3)
ENaC: Epithelial sodium channel
GI: Gastrointestinal
GPCR: G-protein-coupled receptor
IBD: Inflammatory bowel disease
MCT1: Monocarboxylate transporter isoform 1
NBCe1: Electrogenic Na^+^/HCO_3_^−^ cotransporter isoform 1
NBCn1: Electroneutral Na^+^/HCO_3_^−^ cotransporter isoform 1
NHE: Sodium/proton exchanger
NKCC1: Na–K–Cl cotransporter
OSR1: Odd-skipped-related 1
sAC: Soluble adenylyl cyclase
SCFA: Short-chain fatty acids
SLC26: Solute carrier family 26
SPAK: Ste20-related proline alanine-rich kinase
sMCT1: Sodium-coupled monocarboxylate transporter isoform 1
TASK: Two-pore domain potassium channel
TMEM16a: Transmembrane member 16A
TRP: Transient receptor potential cation channel
WNK: With no lysine kinase
wt: Wild type
